# Risks, Benefits, and Molecular Targets of Fenugreek Administration in the Treatment of Hepatocellular Carcinoma

**DOI:** 10.3390/cancers18030458

**Published:** 2026-01-30

**Authors:** Maanya Vittal, Bruna Menegassi, Manlio Vinciguerra

**Affiliations:** 1 School of Pharmacy and Biomolecular Sciences, Liverpool John Moores University, Liverpool L3 3AF, UK; m.vittal@ljmu.ac.uk; 2 Department of Sociology and Communication, Faculty of Social Sciences, University of Salamanca, 37008 Salamanca, Spain; menegassi@usal.es; 3 Research Institute, Medical University Varna, 9002 Varna, Bulgaria; 4 Department of Medicine and Surgery, Libera Universita’ Mediterranea (LUM), 70010 Casamassima, Italy

**Keywords:** fenugreek (*Trigonella foenum-graecum*), hepatocellular carcinoma, diosgenin, trigonelline, molecular targets, apoptosis, hepatoprotection, herb–drug interactions

## Abstract

Fenugreek (*Trigonella foenum-graecum*) has attracted growing interest as a complementary agent in the management of hepatocellular carcinoma (HCC), a leading cause of cancer-related mortality worldwide. Its rich botanical and phytochemical profile, including key bioactive compounds such as diosgenin, trigonelline, saponins, and flavonoids, underpins a spectrum of biological activities relevant to liver cancer therapy. This review critically examines the risks, benefits, and molecular targets of fenugreek administration in HCC, synthesising current evidence on extraction methods, standardisation, pharmacokinetics, and mechanisms of action. Preclinical studies highlight fenugreek’s antitumor efficacy, mediated by apoptosis induction, cell cycle regulation, and modulation of oxidative stress and inflammatory pathways, while its hepatoprotective effects are supported by robust antioxidant and anti-inflammatory properties. However, the safety profile is nuanced, with potential risks including reproductive toxicity, rare hypersensitivity reactions, and herb–drug interactions, particularly in patients with compromised hepatic function or polypharmacy. The review identifies critical gaps in clinical evidence, especially regarding long-term safety and synergistic effects with conventional therapies and underscores the need for rigorous standardisation and patient monitoring. We describe the potential integration of fenugreek into multimodal HCC treatment strategies, if safety concerns are addressed. Future research should elucidate precise molecular targets, optimise formulations, and conduct well-controlled clinical trials to fully realise fenugreek’s therapeutic potential in HCC management.

## Simple Summary

Liver cancer is a serious disease with limited treatment options, and many patients experience side effects from current therapies. Fenugreek is a commonly used medicinal plant that has shown potential benefits for liver health and cancer prevention in laboratory studies. This review explores whether fenugreek could be a useful supportive option in the treatment of liver cancer. The authors aim to summarize current knowledge on how fenugreek and its natural compounds may slow cancer growth, protect liver cells, and interact with key biological processes involved in cancer development. At the same time, possible safety concerns, such as side effects and interactions with cancer drugs, are carefully discussed. By bringing together evidence on benefits, risks, and biological mechanisms, this work highlights both the promise and the limitations of fenugreek. The findings may help researchers identify knowledge gaps, guide future clinical studies, and support safer, more evidence-based use of plant-derived compounds in liver cancer research.

## 1. Introduction

Hepatocellular carcinoma (HCC) remains a leading cause of cancer-related mortality worldwide, presenting a significant challenge due to its aggressive nature and limited therapeutic options. The search for novel, effective, and safer interventions has intensified, with increasing attention directed toward natural products and traditional medicinal plants [1,2]. Among these, fenugreek (*Trigonella foenum-graecum*) has emerged as a candidate of interest, owing to its longstanding use in traditional medicine and its reported antitumor activities [3]. Preclinical studies have demonstrated that fenugreek extracts and their bioactive constituents can exert cytotoxic effects on HCC cell lines, modulate key signalling pathways, and influence the expression of proteins involved in cell proliferation and apoptosis [4].

Despite these promising findings, translating fenugreek’s anticancer potential into clinical practice is hindered by several limitations. The precise molecular mechanisms underlying its effects remain incompletely understood, and the variability in extract composition poses challenges for standardisation and reproducibility. While animal studies generally support a favourable safety profile for fenugreek, there are reports of potential toxic and teratogenic effects, as well as molecular evidence suggesting possible hepatotoxicity and nephrotoxicity at high doses or with prolonged exposure [5,6]. The lack of robust clinical studies further limits our understanding of the true therapeutic window and risk profile of fenugreek in the context of HCC [3]. Recent advances in molecular biology and analytical techniques have facilitated deeper investigations into the multitargeted actions of fenugreek’s bioactive compounds, such as diosgenin, trigonelline, α-tocopherol and quercetin 3-arabinoside, which have been shown to modulate cell cycle regulators and induce apoptosis in cancer cells [7]. Proteomic and transcriptomic analyses are beginning to unravel the complex interactions between fenugreek constituents and cellular pathways, offering new insights into its potential mechanisms of action and safety considerations [8].

This review critically evaluates the current evidence on the risks, benefits, and molecular targets of fenugreek administration for the treatment of hepatocellular carcinoma. We will synthesise findings from preclinical and toxicological studies, discuss the implications of molecular profiling, and highlight knowledge gaps that must be addressed to inform future research and clinical translation. By providing a comprehensive overview, this article seeks to guide the rational development and safe application of fenugreek-based interventions in liver cancer therapy.

## 2. Botanical and Phytochemical Profile of Fenugreek

Fenugreek (*Trigonella foenum-graecum* L.) is an annual herbaceous plant in the Fabaceae family, widely recognised for its trifoliate leaves and slender, hollow stems. Native to the Mediterranean region, fenugreek has been cultivated extensively across Asia and Africa due to its culinary, medicinal, and agricultural importance. Taxonomically, fenugreek belongs to the genus Trigonella, which is distinguished by small, yellowish-white flowers and elongated pods containing numerous seeds. Across various cultures, fenugreek has been valued for its purported health benefits, including its use as a digestive aid, a lactation enhancer, and a treatment for metabolic disorders [9]. Its seeds and leaves play a central role in culinary traditions, especially in South Asian cuisine, and have a longstanding presence in traditional medicine systems such as Ayurveda and Unani.

The phytochemical profile of fenugreek is notably diverse, comprising alkaloids, amino acids, coumarins, flavonoids, saponins, polyphenols, steroids, lipids, carbohydrates, and hydrocarbons [9]. Fenugreek seeds are especially abundant in dietary fibre, phospholipids, glycolipids, oleic acid, linolenic acid, linoleic acid, choline, vitamins (A, B1, B2, C, nicotinic acid, niacin), and galactomannans [10]. Flavonoids and alkaloids are among the most influential contributors to the biological activities associated with fenugreek seeds [11].

Advanced analytical techniques such as HPLC-DAD, LC/MS, GC-MS, and NMR have facilitated the identification of various compound classes, including flavonol glycosides, saponins, particularly steroidal saponins with sugar moieties attached to the C-3 OH position of diosgenin or yamogenin and trigonelline [12,13,14]. Methanolic extracts of fenugreek seeds have revealed a broad spectrum of phytochemical classes, each contributing to distinct biological activities [15].

The leaves and stems of fenugreek also contain notable phytochemicals, such as quercetin, catechin, cinnamic acid, coumaric acid, and a high concentration of soluble fibres [16]. The total phenolic and flavonoid content varies depending on the plant part and processing method, with unprocessed seeds and air-dried leaves retaining high levels. Fenugreek also contains pinitol, and preparative work has led to the isolation of six flavonol glycosides, further enriching its phytochemical diversity [13]. The presence of these compounds supports fenugreek’s antioxidant, hypoglycemic, hypolipidemic, anti-inflammatory, and anticancer activities [12]. Variation in the chemical composition of fenugreek seeds among wild ecotypes points to potential for both medicinal and nutritional applications.

Key bioactive compounds in fenugreek include diosgenin, 4-hydroxyisoleucine, and dietary fibre, which have drawn interest for their physiological effects, especially regarding liver health [17]. These constituents have shown positive effects on liver function, glucose tolerance, inflammation, insulin action, blood lipids, and cardiovascular health [17]. Animal studies have confirmed fenugreek’s hepatoprotective effects, with supplementation restoring altered levels of total and direct bilirubin, ALT, ALP, catalase, and SOD activities in models of thioacetamide-induced liver damage [18]. These protective effects are attributed to the anti-inflammatory, antioxidant, and regenerative properties of fenugreek’s phytochemicals [19].

Fenugreek supplementation has also been found to protect against chemical-induced liver toxicity, such as that caused by dieldrin and carbon tetrachloride, by maintaining haematological, renal, and hepatic biomarkers, reducing lipid peroxidation, and preserving antioxidant enzyme activities [20]. In studies of adriamycin-induced hepatotoxicity, fenugreek seed extract normalised AST and ALT levels, reduced lipid peroxidation, and increased SOD and CAT activities, highlighting its antioxidant properties [21].

Standardised glycoside-based fenugreek seed extracts have demonstrated anti-inflammatory, antioxidant, and anti-fibrotic effects, offering hepatoprotective potential against liver fibrosis in laboratory animals [22]. Fenugreek seed dietary supplementation has also been shown to enhance hepatic antioxidant defence enzyme activities in aged mice [23]. Metabolomic studies reveal that fenugreek influences metabolic pathways in the liver, including carnitine biosynthesis, cholesterol and bile acid metabolism, and arginine biosynthesis, which may contribute to its beneficial effects on liver health [24].

The extensive phytochemical composition of fenugreek, including steroids, alkaloids, saponins, polyphenols, and flavonoids, is thought to be responsible for its disease-preventive and health-promoting effects, particularly at the cellular and molecular levels [25]. Notably, clinical and animal studies have not reported hepatic toxicity at typical doses, and fenugreek has not been associated with liver injury [26,27,28].

Regarding HCC, fenugreek has garnered interest for its antitumor activity. Crude methanol seed extracts have been assessed for anticancer mechanisms using the HepG2 cell line, a widely accepted model for HCC. Although the precise molecular mechanisms underlying fenugreek’s anticancer effects in HCC are not yet fully understood, experimental studies suggest that multiple signalling pathways are involved [3,4]. The in vitro cytotoxicity of fenugreek against cancer cells suggests its potential for cancer prevention and treatment, including HCC, although the protein profile of fenugreek extracts can differ considerably across regional subtypes [8]. Current research emphasises the need for further investigation to clarify the molecular targets and pathways modulated by fenugreek in hepatocellular carcinoma [3].

**Figure 1:**
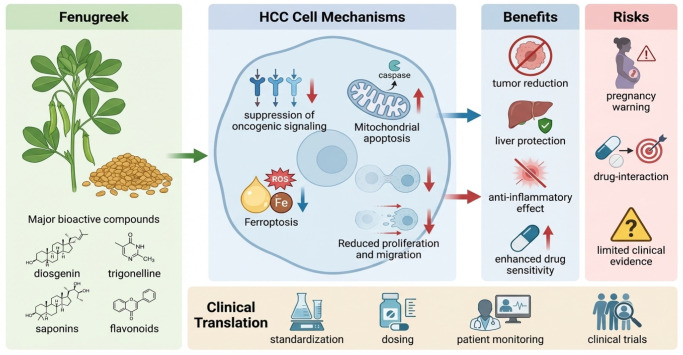
Schematic overview of fenugreek (*Trigonella foenum-graecum*) bioactive compounds in hepatocellular carcinoma (HCC). These compounds modulate oncogenic and stress-response pathways to promote apoptosis, inhibit proliferation and migration, and enhance chemosensitivity in HCC cells. Additionally, they offer hepatoprotective and metabolic benefits. Potential risks such as reproductive toxicity, herb–drug interactions and limited clinical evidence emphasise the need for standardisation and clinical monitoring.

Overall, fenugreek stands out as a botanically and phytochemically rich plant with a long tradition of use and a growing body of evidence supporting its health benefits, particularly for liver health and hepatocellular carcinoma. Its seeds and leaves contain a complex mixture of bioactive compounds, including saponins, flavonoids, alkaloids, and fibres, which together contribute to its antioxidant, anti-inflammatory, and anticancer properties as shown in Figure 1. Although the molecular mechanisms underlying its effects on HCC remain unclear, the current literature supports fenugreek’s potential as a complementary therapeutic agent with a favourable safety profile.

## 3. Extraction Methods and Standardisation of Fenugreek Preparations

The therapeutic potential of fenugreek in HCC is closely linked to its rich phytochemical profile and the efficiency of extraction and standardisation methods used to obtain its bioactive constituents. Extraction techniques are pivotal for determining the yield, purity, and biological activity of fenugreek preparations, which, in turn, influence their efficacy in clinical and experimental settings. Conventional solvent extraction, Soxhlet extraction, microwave-assisted extraction (MAE), maceration, ultrasound-assisted extraction (UAE), and supercritical fluid extraction are among the principal methods employed to isolate bioactive compounds from fenugreek seeds. The choice of solvent, pre-treatment, and optimisation parameters plays a critical role in the efficiency of diosgenin and other key phytochemicals extraction [29]. Comparative studies have demonstrated that UAE generally provides higher extract yields and diosgenin content than MAE and other conventional methods, with yields ranging from 1.04% to 32.48% and diosgenin content between 15.82 mg/100 g and 40.37 mg/100 g of seed powder. For instance, using 80% ethanol, UAE yielded 21.48% extract and 40.37 mg/100 g diosgenin, whereas MAE produced 7.83% extract and 35.50 mg/100 g diosgenin under similar conditions [30]. The type and concentration of solvent, as well as extraction time, are critical factors in optimising extraction efficiency, with aqueous ethanol and methanol frequently used for their ability to extract antioxidant compounds [31].

Emerging technologies such as ultrasound (55.6% of studies), microwave (37.0%), cold plasma (3.7%), and combined approaches have further enhanced fenugreek extract yield and biological activity, with ultrasound being the most effective and widely studied [32]. The use of green solvents, including acetone, ethanol, and water, has been validated for efficient extraction and quantification of target compounds, with UPLC-MS/MS methods providing high accuracy and linearity. Cyclopentyl methyl ether (CPME) has emerged as a superior solvent for fenugreek seed oil extraction, offering higher efficiency, selectivity, and enhanced retention of bioactive compounds compared to traditional solvents like hexane [33].

Accelerated solvent extraction using hydromethanolic solutions and subcritical butane extraction have also been utilised to target specific compound classes, such as phenolics and edible oils, respectively [34,35]. For protein extraction, optimal yields have been achieved at alkaline pH and specific solid-solvent ratios, with distinct protein bands identified in isolates [36]. Selective extraction techniques have also been employed to isolate galactomannan and fibre-rich fractions, which are relevant for both nutritional and therapeutic applications [37,38]. Standardisation of fenugreek extracts is essential to ensure reproducibility, safety, and efficacy, particularly in clinical and pharmacological contexts. High-performance thin-layer chromatography (HPTLC) has been used to standardise fenugreek extracts with trigonelline as a marker compound, demonstrating good linear dependence of peak area on concentration and reliable quantification [39]. High-performance liquid chromatography (HPLC) is widely employed for the quantitative analysis of saponins and flavonoids, with sequential extraction and purification steps enabling the isolation of specific fractions for analysis [40]. The Folin–Ciocalteu method is commonly used to quantify total phenol content in methanol extracts, providing a measure of antioxidant capacity [41]. For trigonelline quantification, green, rapid, and eco-friendly extraction and quantification methods have been developed and validated for use in pharmaceutical, cosmeceutical, herbal, and food products [42]. Self-emulsifying drug delivery systems (SEDDS) containing standardised fenugreek dry extract have been formulated to improve stability and bioavailability, with properties examined by particle size analysis, zeta potential measurements, permeability assays, and cytocompatibility testing [43]. HPLC-UV methods have been applied to quantify diosgenin in aqueous extracts, supporting the preparation and standardisation of extracts for further studies [44]. Spectrophotometric methods have also been validated for simultaneous quantification of trigonelline, diosgenin, and nicotinic acid in various dosage forms, with assessments of linearity, accuracy, and precision [45].

Quality control of fenugreek extracts is achieved through integration of advanced analytical techniques such as HPLC, TGA, and EPR, which allow for comprehensive profiling of seed quality, including assessment of water content, organic compounds, and inorganic metals [46]. These methods provide reproducible data for quality assessment and are essential for determining the viability, quality, and potential agricultural uses of fenugreek seeds [46].

The importance of standardisation cannot be overstated, as it ensures the consistency of fenugreek preparations, which is crucial for both research and therapeutic applications. Standardised extracts allow for reliable assessment of biological activity, safety, and efficacy, particularly when investigating molecular targets in HCC. Fenugreek’s antitumor activity has been demonstrated in vitro using crude methanol extracts in HepG2 cell models, with evidence suggesting involvement of multiple signalling pathways in its anticancer effects [4]. However, the specific molecular targets and their efficacy in HCC require further investigation, as protein profiles of fenugreek extracts can vary considerably between regional subtypes [8]. The integration of advanced extraction and standardisation methods is essential to maximise the therapeutic benefits of fenugreek, ensure the quality and reproducibility of preparations, and facilitate the identification of molecular targets relevant to HCC treatment. Ultimately, the risks and benefits of fenugreek administration for HCC are closely tied to the extraction and standardisation processes. Modern extraction technologies and rigorous standardisation protocols have enhanced the yield, purity, and biological activity of fenugreek preparations, supporting their potential as complementary therapeutic agents in HCC. Continued advancements in extraction and analytical methodologies will further improve the reliability and efficacy of fenugreek-based interventions, paving the way for future clinical applications in liver cancer management.

Overall, a straightforward and standardised pipeline consisting in extraction → enrichment → functional validation logic would thus rely, respectively, on conventional/higher-yield extraction methods (UAE with 80% ethanol, yielding high diosgenin/trigonelline) → selective enrichment (glycoside-based standardised extracts) → in vitro/in vivo validation of the increased bioactive content and enhanced anti-HCC potency (for instance HepG2 apoptosis via diosgenin-rich fractions, or sorafenib synergy via trigonelline, as described in the following sections). A consensus approach would address variability in regional subtypes/protein profiles and emphasise standardisation to achieve reproducible functional outcomes.

## 4. Key Bioactive Compounds Relevant to Liver Cancer: Therapeutic Effects

The antitumor activity of fenugreek in preclinical HCC models has been validated through various in vitro and in vivo investigations [47,48]. Chemical analysis of fenugreek extracts has revealed a variety of bioactive compounds, especially terpenoids and flavonoids, with squalene and naringenin identified as notable constituents with anticancer effects. Fenugreek’s antitumor properties are linked to its ability to modulate key molecular pathways involved in apoptosis and cell cycle regulation, with caspase-3 activation as a central mechanism [4]. Beyond seed extracts, fenugreek seed oil and protein hydrolysates have also shown cytotoxicity against cancer cells, further supporting the therapeutic promise of fenugreek-derived products in oncology. Reviews and experimental data suggest that fenugreek’s anticancer effects are complex, involving changes in cell metabolism, insulin signalling, and possibly the modulation of drug resistance mechanisms [3,49]. While fenugreek’s cytotoxicity against cancer cells is well documented, direct evidence of synergistic effects with conventional chemotherapeutic agents is still limited, and further research is needed to clarify its potential in combination therapies [15,50]. Standardised glycoside-rich fenugreek seed extracts have shown notable anti-inflammatory, antioxidant, and anti-fibrotic actions, protecting against chemically induced liver fibrosis by reducing oxidative and nitrosative stress and modulating nuclear receptor expression [51]. In models of thioacetamide-induced liver injury, fenugreek supplementation restored key biochemical markers of liver function, including bilirubin, alanine aminotransferase (ALT), alkaline phosphatase (ALP), and antioxidant enzymes such as catalase and superoxide dismutase [51]. Notably, fenugreek has not been linked to hepatotoxicity in preclinical studies, highlighting its favourable safety profile.

Histopathological studies in animal models have demonstrated that fenugreek treatment reduces hepatic inflammation and structural damage, effects attributed to its abundance of anti-inflammatory and antioxidant phytochemicals [19]. Fenugreek supplementation boosts the activities of hepatic antioxidant defence enzymes, such as superoxide dismutase, glutathione reductase, and glutathione peroxidase, thereby reducing oxidative stress and supporting tissue regeneration [23]. In toxicological models, including those involving carbon tetrachloride (CCl4), fenugreek pretreatment offers substantial protection to both the liver and the kidney [52]. Fenugreek’s protective effects on the liver also extend to models of alcohol-induced injury, where polyphenol-rich extracts reduce hepatic toxicity by modulating lipid profiles and decreasing collagen deposition [53]. Aqueous fenugreek extracts have been shown to restore antioxidant status and normalise liver enzyme levels after toxic exposure, further supporting their role in liver protection. Fenugreek also affects bile composition by lowering cholesterol and lipid peroxides, increasing bile flow, and extending cholesterol nucleation time, which may reduce the risk of gallstone formation and enhance hepatic function [54]. Clinical data supporting fenugreek’s hepatoprotective effects are emerging: a randomised, placebo-controlled trial assessing hydroalcoholic fenugreek seed extract in nonalcoholic fatty liver disease (NAFLD) indicated possible benefits, but the results were not conclusive, emphasising the need for larger, well-designed studies [55]. Metabonomic analyses have shown that fenugreek supplementation influences hepatic metabolic pathways, including carnitine biosynthesis, cholesterol and bile acid metabolism, and arginine biosynthesis, which may explain its beneficial effects on liver and systemic metabolism [24]. By reducing oxidative stress and inflammatory mediators, fenugreek may influence the tumour microenvironment, potentially improve the effectiveness of other treatments and support liver regeneration. Overall, fenugreek and its key bioactive constituents offer a diverse array of therapeutic effects in the context of HCC.

The therapeutic promise of fenugreek in HCC is rooted in its rich phytochemical profile and the effectiveness of extraction and standardisation techniques, both of which are vital for enhancing the yield and biological activity of its bioactive compounds. Key constituents such as diosgenin, trigonelline, saponins, flavonoids, and proteins have been the focus of extensive research, particularly regarding their anticancer effects in liver cancer. The efficiency of extracting these compounds is closely tied to the chosen method and solvent, with UAE and MAE standing out for their ability to maximise diosgenin content and overall extract yield [30]. The adoption of environmentally friendly solvents and advanced analytical methods, such as UPLC-MS/MS and HPLC, has further enhanced the consistency and reliability of fenugreek extracts, which are critical for both scientific investigation and clinical use [42]. Diosgenin, a steroidal saponin, stands out as the most thoroughly investigated anticancer agent in fenugreek, demonstrating robust activity against HCC cells. It suppresses cell proliferation, triggers apoptosis, and induces cell cycle arrest, especially at the G2/M phase. The proapoptotic action of diosgenin involves the generation of reactive oxygen species (ROS) and activation of the mitochondrial pathway, as shown by nuclear shrinkage, condensation, and fragmentation in HepG2 cells treated with diosgenin [56]. Diosgenin activates caspase-3, -8, and -9, promotes PARP cleavage and cytochrome c release, increases Bax expression, reduces Bid and Bcl-2 levels, and elevates the Bax/Bcl-2 ratio, all indicative of apoptosis [56]. The compound also induces strong ROS production, which may drive apoptosis via ASK1 and subsequent JNK/p38 MAPK activation in HepG2 cells.

Beyond its effects on apoptosis, diosgenin suppresses both constitutive and inducible STAT3 activation, a pathway commonly upregulated in HCC, without impacting STAT5 [57]. This is accomplished by inhibiting upstream kinases such as c-Src, JAK1, and JAK2, and by inducing SH-PTP2, which correlates with STAT3 downregulation [57]. The downstream consequences include diminished expression of STAT3-regulated genes, reduced cell proliferation, and enhanced apoptotic effects when combined with chemotherapeutic agents such as paclitaxel and doxorubicin [44,58]. Diosgenin also interferes with NF-κB/STAT3 signalling, resulting in lower oncogene expression and reduced proliferation in HCC cells. Diosgenin further induces cell cycle arrest in S and G2/M phases and promotes DNA damage, with apoptosis linked to increased BBC3 expression, a crucial factor in the intrinsic apoptosis pathway [59]. The anticancer efficacy of diosgenin is amplified when delivered via nanoparticles or niosomes, which exhibit lower IC50 values and greater cytotoxicity against HCC cells than free diosgenin [60]. Derivatives of diosgenin, including thio(seleno)ureas and glycomimetics, show even greater anti-cancer activity and apoptosis induction in HepG2 cells than diosgenin itself [61].

Additionally, diosgenin inhibits the expression of TAZ, an oncogenic transcription co-activator in the Hippo pathway, indicating another avenue for its anti-HCC effects [62]. In animal studies, diosgenin reduces neutrophil infiltration and enhances mitochondrial health in liver injury, which may help protect against HCC development [63]. Both diosgenin and its glycoside form, dioscin, can reverse multidrug resistance in HCC cell lines, suggesting their potential as adjuncts in chemotherapy [64]. Diosgenin also influences platelet function and coagulation factors, thereby reducing the metastatic potential of HCC in vivo [65]. Trigonelline, another major alkaloid in fenugreek, has shown anti-carcinogenic properties and is considered a promising chemopreventive agent against cancer progression, including HCC [66]. Experimental data indicate that trigonelline impedes the migration of human hepatocarcinoma (Hep3B) cells by downregulating the Raf/ERK/Nrf2 signalling pathway and reducing the activity of antioxidant enzymes, including superoxide dismutase, catalase, and glutathione. It also lowers matrix metalloproteinase 7 (MMP-7) gene expression, further contributing to its anti-migratory effects [66]. Notably, trigonelline enhances the effectiveness of sorafenib, a standard HCC therapy, by facilitating sorafenib-induced ferroptosis in HCC cells through inhibition of NRF2, a central regulator of antioxidant defence [67]. This suggests trigonelline’s potential to enhance pro-ferroptotic treatments in HCC.

Fenugreek is also rich in flavonoids such as naringenin, which have been shown to promote apoptosis and suppress proliferation in HepG2 cells, with mechanisms involving increased expression of p53, Bax, and PCNA, and activation of caspase-3 [4]. Flavonoids are also known for their antioxidant and anti-inflammatory activities, which enhance fenugreek’s anticancer potential [68]. Proteomic studies have shown that the protein composition of fenugreek extracts can vary widely among regional varieties, potentially affecting their cytotoxicity against cancer cells [8]. Selective cytotoxic effects have been observed in vitro across different cancer cell lines, suggesting that the interplay between flavones and saponins may underlie these outcomes [40]. Fenugreek generally exhibits a favourable safety profile, with no evidence connecting it to liver injury [69]. Nonetheless, some reviews have noted toxicological effects, including teratogenicity, in animal models and humans, emphasising the necessity for rigorous standardisation and quality assurance of fenugreek products [5]. The use of advanced extraction and standardisation techniques is crucial for optimising fenugreek’s therapeutic value, ensuring product quality and consistency, and enabling the identification of molecular targets relevant to HCC therapy. Overall, fenugreek administration represents a promising adjunctive strategy for managing hepatocellular carcinoma, with its therapeutic effects closely tied to diosgenin, trigonelline, flavonoids, and other bioactives that influence key molecular pathways involved in tumour development, apoptosis, cell cycle regulation, and drug resistance.

The rich profile of bioactive compounds underpins fenugreek’s preclinical antitumor activity; however, translating this preclinical activity into therapeutic efficacy in HCC requires adequate systemic exposure, hepatic uptake, and intracellular target engagement. The following section examines the pharmacokinetics and metabolism of key constituents (diosgenin, trigonelline, flavonoids), highlighting how formulation strategies enhance bioavailability and potentially amplify molecular effects such as apoptosis induction and pathway modulation in hepatic tissue.

## 5. Pharmacokinetics and Metabolism of Fenugreek Constituents in Hepatic Tissue

The pharmacokinetics of fenugreek and its principal bioactive constituents, diosgenin, trigonelline, saponins, and flavonoids are central to understanding their therapeutic potential and safety in HCC. The absorption, distribution, metabolism, and excretion (ADME) profiles of these compounds determine their bioavailability and efficacy in hepatic tissue, as well as their potential for drug interactions and toxicity. Fenugreek administration has been shown to influence hepatic lipid metabolism primarily by promoting the excretion of cholesterol and bile acids in faeces, rather than by causing hepatic accumulation [69]. This effect is dose-dependent and is associated with increased faecal excretion of cholesterol and bile acids, without notable changes in hepatic triglyceride or cholesterol levels in certain experimental models. Metabonomic analyses reveal that fenugreek supplementation induces changes in multiple metabolic pathways in the liver, including carnitine biosynthesis, cholesterol and bile acid metabolism, and arginine biosynthesis, which may underlie its beneficial effects on hepatic and systemic metabolism [24].

The hepatic metabolism of fenugreek constituents is also relevant to their safety and efficacy. In vitro studies have shown that high concentrations of fenugreek extract can inhibit the activity of cytochrome P450 enzymes CYP2D6 and CYP3A4, which are involved in drug metabolism [70]. However, in vivo studies indicate that fenugreek does not inhibit these enzymes to a notable extent, as evidenced by the lack of substantial changes in the urinary metabolic ratios of dextromethorphan and its metabolites [70]. This suggests that, at physiologically relevant doses, fenugreek is unlikely to cause clinically important drug–drug interactions via CYP2D6 or CYP3A4 inhibition. Among the key bioactive compounds, diosgenin is notable for its hepatic metabolism. Diosgenin and its glycoside, dioscin, are not subject to phase I metabolism in the liver but undergo extensive phase II metabolism, as indicated by their disappearance in the S9 liver fraction [71]. Dioscin can be hydrolysed to diosgenin in the gastrointestinal tract, after which diosgenin is conjugated in the liver, primarily via glucuronidation. Both diosgenin and dioscin have been shown to inhibit CYP3A4 activity in vitro, with diosgenin exhibiting an IC50 of 17 μM, suggesting a potential for drug–drug interactions at high concentrations [71]. Quantitative analysis of diosgenin in pharmacokinetic studies is typically performed using high-performance liquid chromatography (HPLC) and ultra-performance liquid chromatography–tandem mass spectrometry (UPLC-MS/MS), which allows for precise measurement of plasma and tissue concentrations [24,72]. Pharmacokinetic studies in rats have demonstrated that diosgenin formulations, such as nanoparticles or niosomes, can markedly increase their maximum plasma concentration (Cmax) and area under the curve (AUC), indicating improved bioavailability compared to the bulk drug [72,73].

Trigonelline, another major alkaloid in fenugreek, demonstrates good absorption and bioavailability in animal models, with no evidence of hepatotoxicity at doses up to 50 mg/kg daily for 21 days [74]. Trigonelline has been shown to protect liver and kidney function, as evidenced by reductions in serum markers of liver injury and improvements in histological liver architecture [75]. In rats, trigonelline intervention reduced hepatic steatosis and liver damage [76]. Analytical methods, such as hydrophilic interaction liquid chromatography (HILIC) coupled with electrospray ionisation tandem mass spectrometry (ESI-MS/MS), have been validated for the quantification of serum trigonelline [77]. Trigonelline has also been evaluated for its effects on hepatic cytochrome P450 enzymes, with some evidence of interaction with CYP3A4 and CYP2D6 in rat liver microsomes, although the clinical significance remains unclear [78]. Toxicological studies indicate that trigonelline is orally available, safe, and non-mutagenic, with a high LD50 and no acute toxicity in rodents, although human data on chronic exposure are limited [79]. Fenugreek saponins, including furostanol glycosides, have been studied for their pharmacokinetics, tissue distribution, and excretion after oral administration in rats, with a focus on hepatic processing [80]. Flavonoids, another major class of fenugreek bioactives, undergo substantial hepatic metabolism, including hydroxylation and demethylation, which generate metabolites that may contribute to their biological effects [81]. These metabolites are considered bioactive and may play a role in the antioxidant and hepatoprotective effects attributed to fenugreek flavonoids [82]. The metabolic pathways modulated by fenugreek and its constituents have important implications for liver health. Fenugreek supplementation has been shown to restore altered levels of bilirubin, ALT, ALP, catalase, and superoxide dismutase in animal models of liver injury, confirming its hepatoprotective role. It also reduces inflammation and structural alterations in hepatic tissue, attributed to its anti-inflammatory and antioxidant properties [19]. Fenugreek’s hypocholesterolemic effects are mediated by enhanced conversion of cholesterol to bile acids via activation of hepatic cholesterol-7α-hydroxylase, leading to improved bile composition and reduced risk of gallstone formation [54,83].

Differences in the metabolism of fenugreek bioactives may affect their therapeutic efficacy. For example, diosgenin’s bioavailability can be enhanced by formulation strategies, while trigonelline’s favourable ADME profile supports its use as a safe adjunct in liver disease [44,74]. The efficacy of fenugreek’s bioactive compounds is also influenced by their oral bioavailability, minimal CYP inhibition, and ability to penetrate tissues, including the liver and possibly the brain [84]. However, variation in genotype and environmental conditions can lead to differences in the biochemical composition of fenugreek, resulting in inconsistent physiological effects and efficacy [85]. Overall, the pharmacokinetic and metabolic profiles of fenugreek and its principal constituents support their beneficial effects on hepatic metabolism and protection against liver injury, with minimal risk of notable drug interactions or toxicity when used in standardised preparations. The differences in metabolism among the various bioactive compounds highlight the importance of formulation and standardisation to optimise therapeutic efficacy in the context of HCC [86].

## 6. Molecular Targets of Fenugreek in Hepatocellular Carcinoma

Regarding its molecular targets, fenugreek’s anticancer properties in HCC are supported by its capacity to influence cell proliferation and apoptosis pathways. Crude methanol extract from fenugreek seeds has demonstrated cytotoxic effects and induced apoptosis in HepG2 cells in a dose-dependent manner, mediated by upregulation of p53, Bax, proliferating cell nuclear antigen (PCNA), and activation of caspase-3 [4]. Air-dried fenugreek leaves and both unprocessed and germinated fenugreek seeds have exhibited marked anti-proliferative and apoptosis-inducing properties against HepG2, MCF-7, and HCT-116 cancer cell lines, compared to normal VERO cells [87]. Fenugreek seed oil has also demonstrated potent cytotoxicity against cancer cells, as indicated by MTT and NRU assays and morphological studies [88]. Fenugreek extract has been shown to induce autophagy and autophagy-associated cell death in Jurkat cells, in addition to its established apoptotic effects, suggesting that autophagy may represent an additional mechanism underlying its anticancer properties [89]. Proteomic analysis has revealed that the protein profile of certain fenugreek extracts differs markedly from other regional subtypes, and in vitro studies have demonstrated notable cytotoxicity of fenugreek against cancer cells, supporting its potential role in cancer prevention and therapy [8]. The anticancer activity of fenugreek is linked to multiple signalling pathways, but the precise efficacy and mechanisms remain unclear [13]. Diosgenin, a major sapogenin in fenugreek, has demonstrated notable anticancer activity and is of particular interest for the treatment of various cancers, including HCC. Trigonelline also shows promise for therapeutic applications in cancer treatment [90].

Flavonoids, abundant in fenugreek, are recognised for their antioxidant, anti-inflammatory, and anticancer properties, and have been shown to exert cytotoxic effects on various cancer cell lines, including those relevant to HCC. Flavonoids from fenugreek and other sources have been shown to inhibit cancer cell proliferation, induce apoptosis, and suppress metastasis in HCC models. Fenugreek’s anti-inflammatory and antioxidant properties further support its hepatoprotective effects. Histological analysis in animal studies has shown that fenugreek treatment leads to a marked reduction in inflammation and structural changes in the liver, effects attributed to its rich phytochemical content with anti-inflammatory, antioxidant, and regenerative capacities [19]. Supplementation with fenugreek seed extract in models of liver injury resulted in normalisation of liver enzymes, reduction in lipid peroxidation, and increased activities of antioxidant enzymes such as SOD and CAT, indicating a beneficial impact on hepatotoxicity due to its antioxidant effect [21,91]. Fenugreek seed dietary supplementation has also been shown to enhance hepatic antioxidant defence enzyme activities in aged mice [23]. With respect to drug resistance, fenugreek has been shown to modulate CYP3A4, an enzyme involved in the metabolism of drugs such as ribociclib, highlighting the potential for herb–drug interactions in oncology [92]. However, both in vitro and in vivo studies indicate that fenugreek may not have a substantial impact on the metabolic activity of CYP2D6 and CYP3A4. There is no direct evidence linking fenugreek to the modulation of ABCB1 (P-glycoprotein) or to the reversal of drug resistance in HCC via ABCB1, CYP3A4, or CYP2D6, in pharmacokinetic studies [93].

Overall, fenugreek and its principal constituents provide beneficial effects on hepatic metabolism and protection against liver injury, with minimal risk of major drug interactions or toxicity when used in standardised preparations. Diosgenin, trigonelline, saponins, and flavonoids contribute to fenugreek’s anticancer and hepatoprotective actions, acting through modulation of cell proliferation, apoptosis, antioxidant defence, and anti-inflammatory pathways. The differences in metabolism among the various bioactive compounds underscore the importance of formulation and standardisation to optimise therapeutic efficacy in HCC. Although the precise molecular targets and mechanisms remain to be fully elucidated, current evidence supports the relevance of fenugreek’s bioactives in the prevention and adjunctive treatment of hepatocellular carcinoma.

## 7. Risks and Safety Profile of Fenugreek Use in Liver Cancer Patients

A comprehensive evaluation of Fenugreek risks, benefits, and molecular targets is essential for informed clinical translation, particularly in the context of liver cancer therapy, where hepatic function is already compromised. Toxicological assessments of fenugreek have revealed a complex safety landscape. Animal studies have demonstrated that fenugreek seed extracts can exert antifertility effects, with notable impacts on sperm parameters, oxidative status, and testicular histoarchitecture in rodents. These changes have translated into reduced fertility indices and adverse pregnancy outcomes in animal models, suggesting a potential risk for reproductive toxicity [94]. In humans, fenugreek consumption during pregnancy has been associated with congenital malformations, including hydrocephalus, anencephaly, and spina bifida, as well as increased fetal death rates and morphological abnormalities, raising concerns about its teratogenic potential [95]. Beyond reproductive effects, gastrointestinal disturbances such as diarrhoea, flatulence, nausea, and vomiting have been reported in adults, and rare but severe cutaneous adverse reactions, including toxic epidermal necrolysis, have been documented [96]. Despite these adverse findings, standardised fenugreek seed extracts have demonstrated a broad margin of safety in toxicological studies. No observed adverse effect levels have been established at doses up to 1000 mg/kg body weight per day in rats, with no evidence of mutagenicity or genotoxicity in standard assays [97,98,99]. Clinical and animal studies have not identified notable adverse effects on body weight, food and water consumption, organ weights, haematological parameters, or clinical biochemistry at therapeutic doses [100,101]. Nevertheless, the potential for rare but severe hypersensitivity or toxic reactions persists, particularly in sensitive populations. The risk of herb–drug interactions is a critical consideration in oncology, where polypharmacy is common and hepatic metabolism is often compromised. A notable case involved a patient with metastatic breast cancer who developed grade III hepatotoxicity while receiving ribociclib, a CDK4/6 inhibitor, in combination with a fenugreek-based supplement. The temporal association and subsequent normalisation of liver enzymes upon discontinuation of both agents suggested a probable herb–drug interaction, as assessed by established causality scales [102]. Fenugreek is known to modulate cytochrome P450 enzymes, particularly CYP3A4, which is responsible for the metabolism of many anticancer agents, including ribociclib. This interaction underscores the need for vigilance when fenugreek is used concomitantly with drugs metabolised by hepatic enzymes, as it may alter drug pharmacokinetics and increase the risk of toxicity or therapeutic failure [95,102]. The impact of fenugreek on hepatic metabolism and the potential for hepatotoxicity is nuanced. On one hand, fenugreek supplementation has demonstrated robust hepatoprotective effects in preclinical models of chemically induced liver injury. These benefits are attributed to its antioxidant properties, which help maintain biochemical parameters and restore altered levels of liver enzymes and antioxidant activities [20,52,91]. In models of Adriamycin-induced hepatotoxicity, fenugreek normalised liver enzyme levels and reduced lipid peroxidation, further supporting its protective role [21]. Fenugreek also modulates hepatic metabolic pathways, including carnitine biosynthesis, cholesterol and bile acid metabolism, and arginine biosynthesis, which may contribute to its beneficial effects on liver function and systemic metabolism [24]. Additionally, fenugreek administration increases the excretion of cholesterol and bile acids and inhibits hepatic lipid accumulation by promoting faecal lipid excretion [69]. Conversely, there are documented cases of fenugreek-induced hepatotoxicity, particularly at high doses or in specific contexts. Some studies have reported increased hepatic enzyme levels, reduced total protein and albumin, upregulation of pro-apoptotic markers, and impaired hepatic histology following fenugreek intoxication, indicating potential for liver injury [103]. In vitro, fenugreek extract inhibits CYP2D6-mediated metabolism, but in vivo studies suggest it does not inhibit CYP2D6 or CYP3A4 activity to a notable extent, highlighting a discrepancy between experimental models and clinical relevance. Nevertheless, the possibility of herb–drug interactions and hepatotoxicity cannot be excluded, particularly in patients with pre-existing liver disease or those receiving polypharmacy [5,99,104].

From a clinical translation perspective, the use of fenugreek in liver cancer therapy necessitates careful patient selection and monitoring. The potential for reproductive toxicity and teratogenicity precludes its use in pregnant women and those planning conception. The risk of herb–drug interactions, particularly with agents metabolised by CYP3A4 and CYP2D6, mandates thorough medication reconciliation and patient education. Regular monitoring of liver function tests is advisable during fenugreek supplementation, especially in individuals with underlying hepatic impairment or those receiving hepatotoxic drugs. Healthcare providers should maintain a high index of suspicion for adverse reactions and counsel patients regarding the signs and symptoms of liver injury and hypersensitivity. Overall, fenugreek exhibits a dualistic profile, offering hepatoprotective and antitumor benefits while posing risks of reproductive toxicity, rare hypersensitivity reactions, and potential hepatotoxicity, particularly in susceptible populations or when used concomitantly with drugs. Its impact on hepatic metabolism and drug interactions underscores the necessity for individualised risk assessment and vigilant clinical monitoring in the context of HCC management. The current evidence supports the cautious integration of fenugreek into therapeutic regimens for liver cancer, with a clear emphasis on patient safety, informed consent, and ongoing pharmacovigilance. These safety considerations, along with pharmacokinetic variability and potential drug–herb interactions, highlight key translational challenges for fenugreek use in patients with liver cancer (Table 1).

## 8. Proposed Preclinical-to-Clinical Translational Roadmap for Fenugreek in HCC

To offer a translational roadmap for Fenugreek in HCC, we propose a preclinical-to-clinical framework supported by pharmacodynamic biomarkers. This framework may outline a stepwise progression:

(a) Preclinical stage: Extraction/standardisation → in vitro functional validation (HepG2/Hep3B cytotoxicity, apoptosis/ferroptosis assays) → in vivo hepatoprotective/antitumor efficacy (DEN-induced HCC models, xenograft models) with candidate pharmacodynamic (PD) biomarkers (tissue levels of Bax/Bcl-2 ratio, cleaved caspase-3, NRF2 downregulation, ROS generation, or phospho-STAT3 reduction).
(b) Translational/early clinical stage: Phase I dose-escalation studies in advanced HCC patients (focus on safety, pharmacokinetics, and PD biomarker modulation in liver biopsies or blood).
(c) Clinical validation stage: Phase II proof-of-concept trials assessing objective response rate (ORR), progression-free survival (PFS), and biomarker correlates; eventual Phase III adjuvant/combinatorial designs.

Finally, we emphasise the urgent need for PD biomarkers (e.g., circulating miRNAs linked to apoptosis or ferroptosis, and serum trigonelline/diosgenin levels) to bridge preclinical promise and clinical endpoints, as no dedicated HCC clinical trials for fenugreek currently exist.

## 9. Beneficial Effects of Fenugreek on HCC-Associated Comorbidities: Oxidative Injury and Microbial Infections

HCC frequently co-occurs with comorbidities that accelerate disease progression, notably oxidative injury (driven by chronic inflammation, viral hepatitis, alcohol, or metabolic dysregulation) and microbial infections (bacterial translocation in cirrhosis, persistent viral infections, or opportunistic pathogens amid immunosuppression). Fenugreek and its bioactive constituents (diosgenin, trigonelline, flavonoids, polyphenols) exert protective effects against these through potent antioxidant, anti-inflammatory, and antimicrobial activities. Oxidative injury is central to HCC pathogenesis, promoting DNA damage, lipid peroxidation, and transition from chronic liver disease to malignancy. Fenugreek mitigates this by bolstering hepatic antioxidant defences. In ageing mice, dietary supplementation with fenugreek seeds significantly upregulated superoxide dismutase (SOD), glutathione peroxidase (GPx), and glutathione reductase (GR) activities, reducing reactive oxygen species (ROS) accumulation and lipid peroxidation in the liver [23]. Polyphenolic fenugreek seed extracts protected against ethanol-induced hepatic collagen and lipid accumulation by restoring SOD and catalase levels while decreasing malondialdehyde (MDA), with effects comparable to silymarin [110]. Fenugreek also attenuated cisplatin-induced ROS generation and MDA elevation while enhancing total antioxidant capacity [111]. Diosgenin suppresses ROS-mediated pathways (e.g., ASK1/JNK/p38 MAPK) and supports mitochondrial integrity in HCC models, alleviating oxidative burden [56]. Trigonelline inhibits NRF2-dependent antioxidant enzymes in stressed cells, modulating redox balance protectively without favouring tumour survival [111].

Fenugreek further offers antimicrobial benefits relevant to HCC-associated infections. Its broad-spectrum activity (antibacterial, antifungal, antiviral) stems from polyphenols, saponins, and alkaloids, which disrupt microbial membranes and limit inflammation [112,113]. Preclinical studies confirm efficacy against pathogens like *Staphylococcus aureus*, *Escherichia coli*, and *Candida albicans* [85,91,114]. In cirrhosis-related contexts, where gut dysbiosis and bacterial translocation fuel infections and endotoxin-driven progression, fenugreek-enriched diets increased beneficial bacteria such as *Akkermansia muciniphila*, reducing metabolic inflammation and NAFLD-like changes overlapping with HCC comorbidities [115]. This modulation of the gut-liver axis may indirectly lower infection risk. Collectively, fenugreek’s antioxidant restoration, ROS scavenging, and antimicrobial support position it as a complementary agent for managing oxidative injury and infection-related comorbidities in HCC. Preclinical data are robust, but clinical validation in HCC patients is needed to confirm efficacy and safety amid polypharmacy.

## 10. Conclusions

Current evidence highlights fenugreek as a promising botanical with multifaceted anticancer and hepatoprotective properties relevant to hepatocellular carcinoma, acting through the modulation of oxidative stress, inflammation, apoptosis, and cell proliferation [3]. Its bioactive constituents, including trigonelline and various saponins, have demonstrated cytotoxicity against cancer cells and low toxicity in preclinical models, supporting its potential as a preventive and therapeutic agent [8,116]. However, the molecular mechanisms underlying these effects remain incompletely defined, with proteomic analyses revealing substantial variability in extract composition and activity depending on source and preparation [8]. Despite encouraging laboratory data, clinical studies in HCC patients are lacking, and concerns persist regarding potential toxicity, herb–drug interactions, and the need for rigorous standardisation [3,4]. To advance the clinical utility of fenugreek in liver cancer, future research should prioritise well-designed trials, comprehensive safety assessments, and mechanistic studies to clarify its molecular targets and optimise its integration into oncological care [3]. It would therefore be important to place fenugreek as a potential multimodal complementary agent in HCC management, integrating its hepatoprotective effects, direct antitumor mechanisms, and adjuvant potential. In this respect, rigorous standardisation of extracts and formulation optimisation, and phase I/II clinical trials in well-defined HCC populations with pharmacodynamic biomarkers (serum markers of apoptosis, oxidative stress) should be prioritised.

## Figures and Tables

**Table 1:** Knowledge Gaps, Translational Challenges, and Future Research Priorities for Fenugreek Use in Hepatocellular Carcinoma.

Research Domain	Current State of Evidence	Key Limitations/Unresolved Questions	Proposed Future Directions	Key References
Clinical efficacy in HCC	Strong in vitro evidence (HepG2, Hep3B); limited in vivo tumour models	No randomised clinical trials in HCC patients; unclear clinical benefit	Phase I/II trials evaluating safety, dosing, and efficacy in HCC patients	[105]
Dose standardisation	Wide variability in extracts, solvents, and bioactive content	Lack of consensus on therapeutic dose; batch-to-batch inconsistency	Development of marker-based standardised extracts (e.g., diosgenin, trigonelline)	[106]
Long-term safety	Generally safe in short-term animal and human studies	Limited chronic toxicity data; reproductive and teratogenic risks	Long-term toxicity and reproductive safety studies under clinically relevant dosing	[4]
Herb–drug interactions	In vitro CYP3A4 and CYP2D6 inhibition reported	Clinical relevance unclear; rare but serious hepatotoxicity cases reported	Dedicated interaction studies with sorafenib, ribociclib, and immunotherapies	[107]
Tumour microenvironment effects	Antioxidant and anti-inflammatory actions are demonstrated	Impact on immune cells, fibrosis, and angiogenesis is poorly defined	Studies on immune modulation, macrophage polarisation, and stromal interactions	[78]
Resistance mechanisms	Diosgenin and dioscin reverse multidrug resistance in vitro	Limited evidence in HCC-specific resistance models	Evaluation in sorafenib-resistant and ferroptosis-resistant HCC models	[28]
Bioavailability and delivery	Poor oral bioavailability of diosgenin	Clinical translation is limited by low systemic exposure	Nanoparticles, niosomes, and SEDDS-based delivery systems	[28]
Molecular target specificity	Multiple pathways affected (STAT3, NF-κB, NRF2, Hippo)	Difficult to define primary vs. secondary targets	Multi-omics and target validation studies (CRISPR, phosphoproteomics)	[108]
Population variability	Ecotype- and region-dependent phytochemical variation	Inconsistent biological activity between preparations	Chemotype classification and geographic standardisation	[109]

## Data Availability

No new data were created or analysed in this study.
